# Risk Factors Identification of Unsafe Acts in Deep Coal Mine Workers Based on Grounded Theory and HFACS

**DOI:** 10.3389/fpubh.2022.852612

**Published:** 2022-03-17

**Authors:** Li Yang, Xue Wang, Junqi Zhu, Zhiyuan Qin

**Affiliations:** School of Economics and Management, Anhui University of Science and Technology, Huainan, China

**Keywords:** coal miners, unsafe acts, coal mine accidents, grounded theory, HFACS model

## Abstract

The risk factors affecting workers' unsafe acts were comprehensively identified by Human Factors Analysis and Classification System (HFACS) and grounded theory based on interview data and accident reports from deep coal mines. Firstly, we collected accident case and field interview data from deep coal mines issued by authoritative institutions. Then, the data were coded according to grounded theory to obtain relevant concepts and types. The HFACS model was used to classify the concepts and categories. Finally, the relationship between core and secondary categories was sorted out by applying a story plot. The results show that risk factors of unsafe acts of deep coal mine workers include environmental factors, organizational influence, unsafe supervision and unsafe state of miners, and the main manifestations of unsafe acts are errors and violations. Among them, the unsafe state of miners is the intermediate variable, and other factors indirectly affect risky actions of coal miners through unsafe sates. Resource management, organizational processes and failure to correct problems are the top three risk factors that occur more frequently in unsafe acts. The three most common types of unsafe act are unreasonable labor organization, failure to enforce rules, and inadequate technical specifications. By combining grounded theory and the HFACS framework to analyze data, risk factors for deep coal miners can be quickly identified, and more precise and comprehensive conceptual models of risk factors in unsafe acts of deep coal miners can be obtained.

## Introduction

As one of the most important global energy sources, coal plays a vital role in the world's energy structure. Still, the frequent occurrence of coal mining accidents dramatically threatens the safety of coal production and workers' lives. Studies have shown that more than 90% of coal mine accidents are caused by human factors. These factors often manifest in various unsafe behaviors of people (1, 2). At the same time, with the continuous deepening of coal mining depth, the risk factors inside and outside the mine impacting workers' unsafe acts become complex and diverse. Once workers engage in risky behaviors, it may lead to a series of hazardous events and then to significant coal mining accidents. Therefore, it is an essential prerequisite to ensure safe production in deep coal mines to identify the risk factors of dangerous acts of deep coal miners and reduce the occurrence of unsafe behaviors.

Recently, scholars have carried out studies on the influencing factors of coal miners' unsafe acts. For example, Kapp et al. (3) studied the impact of fatigue on workers' dangerous acts, pointing out that it harms workers' safety performance. Workers are more prone to make mistakes under a fatigued state. Ren et al. (4) also pointed out that with the increase of physical consumption of coal miners, their enthusiasm and efficiency of work decreased significantly. Aliabadi et al. (5) point out that organizational deficiencies are the leading cause of accidents in the mining industry and directly correlate with workers' safety violations and errors. In addition, Li et al. (6) pointed out that the safety attitudes of coal miners positively impact preventative behaviors. Employee attitudes have a significant effect on safety performance, and a good attitude can improve the industrial safety performance of an entire organization. Yu et al. (7) also pointed out that the psychosocial safety climate can reduce miners' risky behaviors through the mediating effect of job stress and burnout. In addition to analyzing the influence of a single or several factors on miners' risky behaviors, scholars investigated multiple risk factors on miners' hazardous actions from a systematic perspective. For example, Wang et al. (8) pointed out that individual perception, environmental support, organizational management system, and experience components are significantly correlated with the unsafe behaviors of coal miners and suggested that young, inexperienced coal miners are more likely to engage in safe behaviors. Yu et al. (9) also used ANP (Analytic Network Proces) and system dynamics models to analyze the influence of individual and group factors, physical environment, safety leadership, and risk management factors on coal miners' unsafe behaviors ranked their importance. In addition, Based on the HFACS model and SEM model, Liu et al. (10) analyzed the influencing factors of coal mine workers' unsafe behaviors. They pointed out that the impact of the external environment, inadequate leadership, preconditions of unsafe behaviors, and organizational influence on workers' dangerous behaviors weakened successively. Fa et al. (11) also used HFACS models, text segmentation technology, and Apriori association algorithms to study risk factors related to coal mine workers' unsafe behaviors from the perspective of individuals and organizations. Their findings indicated that external and organizational influences, inadequate supervision, and dangerous behavior preconditions were the main factors affecting coal mine workers' unsafe behaviors.

Previous research has been enlightening that the study on the risk factors of deep coal mine workers' unsafe act needs to be carried out from many aspects, such as individual workers, organization, management, and environment. The risk factors affecting the unsafe act of deep coal miners are complex and diverse, and few scholars systematically analyze them from multiple levels. Although some scholars have used the HFAC model to analyze the risk factors of the unsafe act of miners, the risk factors are not comprehensive, and there is a lack of analysis of the unsafe state of workers (11, 12). Therefore, it is a challenge to capture the systemic factors that influence the risky act of deep coal miners, which is necessary to adopt systematic thinking and appropriate methods to accomplish this task. HFACS framework offers advantages in a systematic analysis of the role of human factors in accidents, which has been successfully applied in coal mine safety management (10, 11). The framework can be used to explore the unsafe actions of front-line miners and their preconditions and analyze the impact of organizational influence, inadequate supervision, external environment, and other hazardous conditions on human factors. It is a comprehensive and in-depth cause analysis model with powerful applications in determining the human factors in accidents and formulating preventive measures (13). Although human factors were divided under the original HFACS framework, it remained impossible to observe primary and secondary factors or detect any causal relationships. Thus, underlying risk factors for workers' unsafe behaviors could not be comprehensively analyzed. The HFACS and grounded theory combination provide a complementary advantage in risk factor identification. The grounded theory identifies specific risk factors and clarifies their relationships (14). However, factors identified by grounded theory alone may have overlapping concepts and different scales, and the HFACS compensates for this defect. Therefore, the combination of grounded theory and HFACS creates the conceptual model of the risk factors for hazardous actions by deep coal miners and makes abstract and hierarchical relationships between them more apparent. Although some scholars have used grounded theory and the HFACS model to classify and analyze the human error of various risk accidents, this method has not been applied to identify and analyze unsafe risk factors of workers in deep coal mines (12, 15). In addition, coal mine accident reports issued by authoritative institutions at home and abroad are usually used to preliminarily identify the risk factors of unsafe acts of coal miners. Currently, scholars worldwide also use this report to conduct statistical analysis on coal mine accident data (14, 16, 17). Scholars used the HFACS model and coal mine accident report to study the core of coal mine workers' unsafe behavior, mainly focusing on human factor identification, accident cause analysis, and control measures formulation. However, there remains a deficit of research literature on the unsafe act of deep coal mine workers, making it challenging to analyze the potential causes of deep coal mine accidents.

Based on the above analysis, the purpose of this study is to use grounded theory and the HFACS model to analyze the interview data of deep coal mine workers and coal mine accident reports to identify the risk factors affecting workers' unsafe acts. The HFACS model combined with grounded theory can accurately identify the human factors in coal mine accidents and find out the causes of hazardous behaviors of coal miners. It is hoped that this study can improve the depth and breadth of accident analysis methods of deep coal mines, clarify the causes of unsafe acts of deep coal miners, and provide a theoretical basis for formulating intervention strategies. The Improved HFACS-CM model constructed in this study can point out various risk factors of unsafe acts of deep coal miners and point out the interaction of factors at different levels. It can also point out how various factors of human factors ultimately lead to coal mine accidents. The research results of this paper enrich the research methods of unsafe behavior of deep coal miners and lay a particular theoretical foundation for future scholars in this field. Since the research of this paper is based on the accident report of deep coal mines and the interview of deep coal mine workers, the research results of this paper have more practical guiding value for the prevention and control of unsafe acts of deep coal mine workers. Based on the Improved HFACS-CM model constructed in this study, coal mine safety managers can deeply understand the causes of workers' unsafe acts and how human factors lead to coal mine accidents. Based on this, they can establish more effective measures to prevent and control workers' unsafe acts to reduce the occurrence of workers' unsafe acts and coal mine accidents.

## Method

### HFACS Model

#### HFACS Original Model

Shappell and Wiegmann established the HFACS model based on the Swiss cheese model (18). This model identifies the vulnerabilities in cheese and has been widely used in various fields (13). In the chemical industry, based on the HFACS framework, Wang et al. (19) obtained a new model, HFACS-CSMEs (Human Factor Analysis and Classification System for Chemical Small and Medium-sized Enterprises), which can effectively identify and distinguish the causes of chemical accidents, providing a new idea for accident prevention of small and medium-sized chemical enterprises. In the power industry, based on the HFACS framework and fuzzy analytic hierarchy process, Karthick et al. (20) analyzed the internal human factors affecting the operators' performance in nuclear power plants. They pointed out that the key factors leading to human error were cognitive and organizational. In public health, Bickley et al. (21) applied the improved HFACS model to public health to reduce potential errors at different levels in public health systems. Tang et al. (22) proposed an enhanced HFACS personalized safety management model to analyze the impact of human error on construction accidents. Their results indicated that the model was superior to the traditional safety management model. Yildiz et al. (23) pointed out that the transportation industry can employ the HFACS-PV (Human Factor Analysis and Classification System for Passenger Vessel) structure for continuous analysis of ship accidents and qualitative and quantitative analysis combined with other methods. The application of the HFACS model in the mining industry has been relatively mature. For example, Liu et al. (24) conducted a qualitative and quantitative evaluation of major coal mine accidents based on the AHP (Analytic Hierarchy Process) and HFACS-CM (Human Factor Analysis and Classification System for China's Mines) models. They systematically studied the adverse safety behaviors of coal miners and other related factors. Based on the HFACS and SEM (Structural Equation Model) models, Liu et al. (10) analyzed the influencing factors of coal mine workers' risky behaviors. They pointed out that the degree of influence of the external environment, negligent leadership, preconditions of unsafe behaviors and organizational influence on workers' unsafe acts weakened successively. Based on the HFACS model, text segmentation technology, and Apriori association algorithm, Fa et al. (11) studied the factors influencing the unsafe behavior of coal miners from the perspective of individual and organizational factors. They pointed out that external influence, organizational influence, negligent supervision, and risky behavior prerequisite conditions were the main factors influencing the unsafe behavior of coal miners.

**Figure 1:**
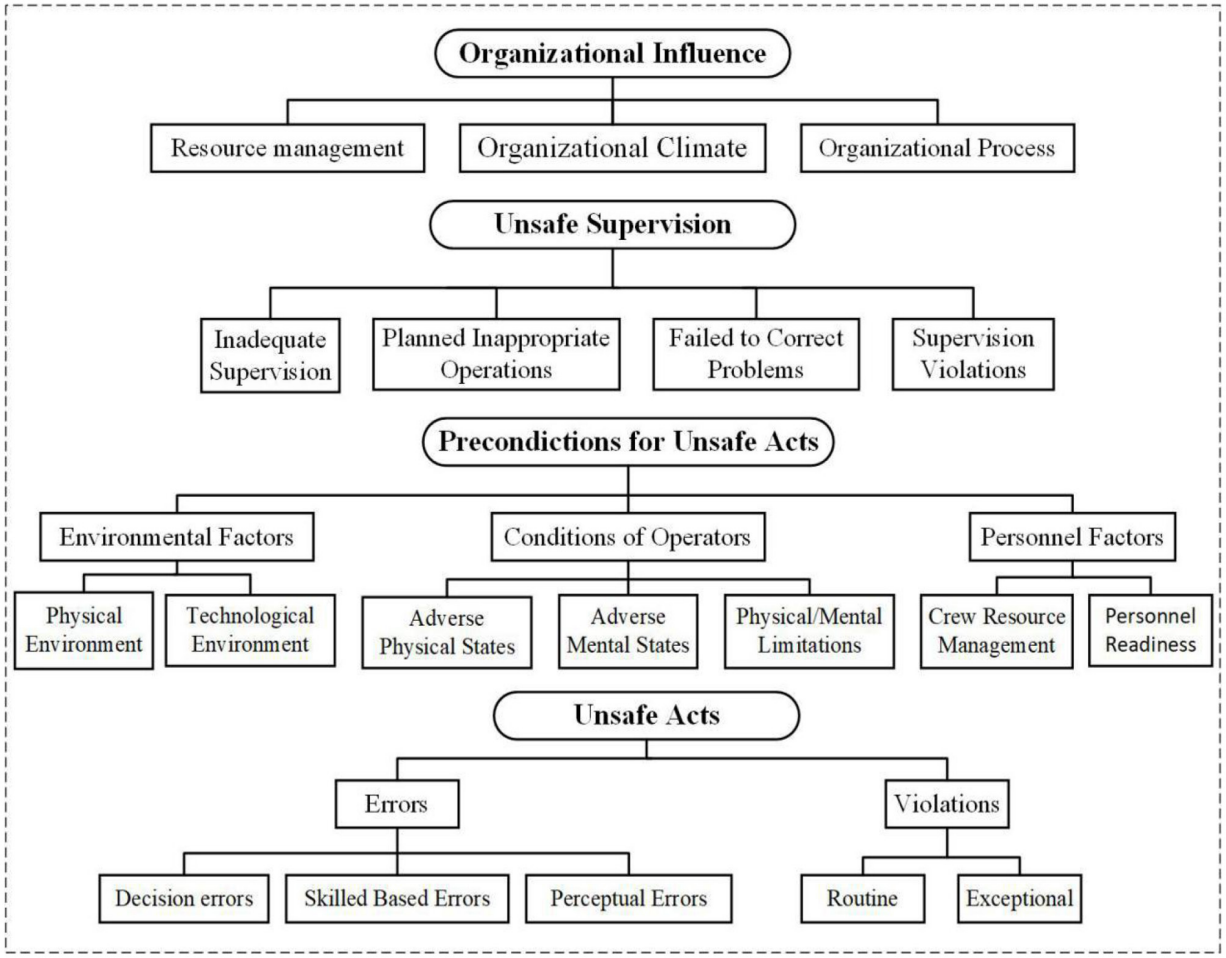
The original HFACS framework.

In the original HFACS model, human error is divided into four levels (25). From high to low, these levels are organizational influence, unsafe supervision, preconditions for unsafe acts, and unsafe behaviors. Each level is divided into several sub-levels, whose definition is used to classify identified causal factors (26). The four levels comprised e19 factor categories, as shown in Figure 1. The original HFACS model did not require expert advice in classifying accident causes and causal factors. Thus, researchers who have mastered the major structures and infrastructure can gradually delineate the occurrence of accidents (27). Explanations of the various levels of the original HFACS model are described below.

The first layer is organizational influence, which refers to the weak organizational framework, defects, lack of team culture, and other factors, laying a foundation for accidents. This level is divided into three subcategories: resource management, organizational climate, and organizational processes. Resource management refers to misguided decisions made by an enterprise in terms of workforce, equipment, and investment, such as improper staffing, lack of education and training, and insufficient equipment and investment. Organizational climate refers to the working environment of an organization, which is defined as the internal factors that affect individual performance, such as teamwork and communication. Finally, organizational processes include internal administrative factors such as formal processes, methods, and oversight, for example, work commitments, reward and punishment systems, and safety procedures.

The second layer is unsafe supervision, which refers to poor operation habits, worker process mistakes, and behaviors that violate rules set by the appropriate supervision and management system. This layer primarily includes inadequate supervision, planned inappropriate operations, failed to correct problems, and supervision violations. Inadequate supervision refers to the failure of work and performance supervision of workers. Planned inappropriate operations are defects in the pre-set operation plan, such as unreasonable labor organization and ineffective implementation of risk response measures. Failure to correct problems refers to management personnel's negligence of effective preventive measures against the improper operation of workers, equipment defects, management errors, and other issues. Finally, supervision violations refer to workers' behaviors who disregard the supervision and management systems, for example, allowing unqualified people to work in high-risk mines.

The third layer is the precondition for unsafe acts, which describes the psychological and physiological states and operational skills that cause hazardous actions. This level includes three sub-categories: environmental factors, conditions of operators, and personnel factors. Environmental factors refer to the factors that adversely affect worker productivity, including the physical and technical environments. Physical environment refers to physical factors such as temperature, noise, and lighting. Technical environment refers to worker performance factors, including equipment, mining design, safety monitoring systems, among others. Operator conditions refer to psychological and physiological states that negatively affect the individual performance of workers. Finally, personnel factors are bad decisions due to a lack of coordination among team members and inadequate personal preparation, for example, a lack of safety equipment.

The fourth layer is unsafe acts, which refers to personally risky actions. There are two types of unsafe acts: errors and violations. Errors refer to unintentional actions, divided into three types: decision, skill, and perceptual errors. Violations refer to intentional disregard of rules and regulations, divided into regular and exceptional types. Regular or routine violations are those tolerated by managers, such as eliminating work steps, risky operations, and disregarding rules and regulations. Exceptional violations refer to those that occur under particular circumstances. These violations are unusual because they occur outside regulations and laws, such as the production organization during the COVID-19 (Corona Virus Disease 2019) pandemic.

#### Improved HFACS-CM Model

Although the original HFACS model factors are an excellent fit, some of them do not fully apply to components of risk associated with unsafe acts of deep coal miners. The model should be improved according to the characteristics of the mining industry and the specific accident site situation (24, 28). The purpose of this paper is to identify the risk factors affecting the unsafe act of miners. The unsafe act layer in the HFACS model refers to the hazardous behaviors of coal mine employees, including front-line workers and related management personnel. Higher-level problems in the model can lead to lower-level problems and risky worker behaviors. Therefore, the HFACS model is suitable for comprehensively identifying risk factors for unsafe acts of deep coal miners. The unsafe act layer in the model has been modified to the unsafe act of the deep coal mine workers to make the model more suitable for this study.

**Figure 2:**
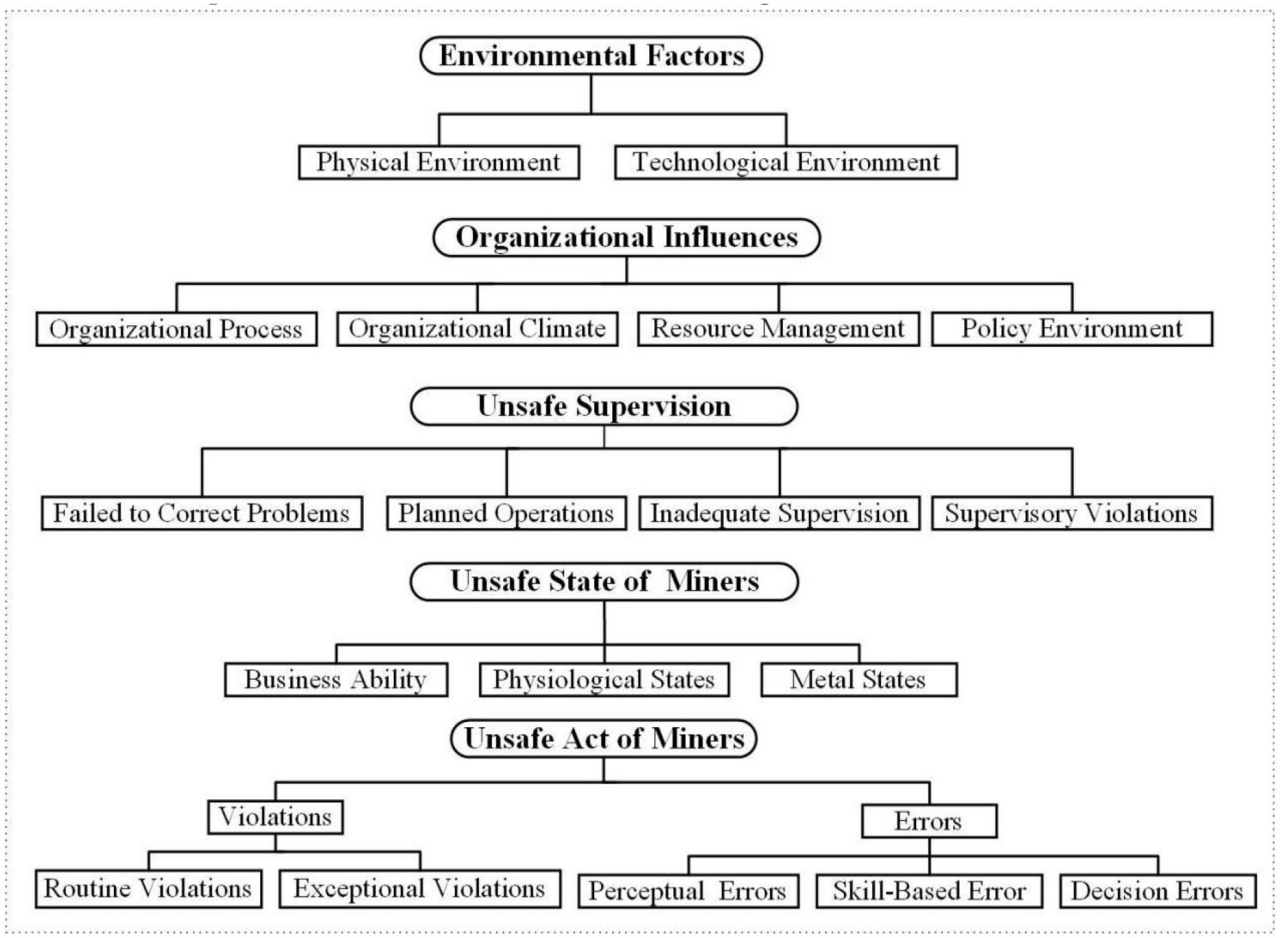
The improved framework of HFACS-CM.

In deep coal mining, physical conditions, such as geological structure, temperature, humidity, and other environmental characteristics, significantly influence workers' unsafe behaviors. In addition, with the continuous progress of mining technology and the rapid development of society, coal miners' behaviors are not only affected by internal factors and the external environment, such as information technology, economy, and politics. Therefore, in this study, the environmental factors included in the preconditions of unsafe acts in the original model are moved to the first layer. The new level (Level 1) consists of the physical, technical, and policy environments, which are summarized as environmental factors. In real-world coal mine production, the premise of unsafe acts in the original model is changed to the unsafe state of miners, including workers' mental and physiological states and business ability. Achieving coal mine production objectives requires cooperation and communication between team members. Therefore, this study adds teamwork and communication to the original HFACS model and classifies them within the organizational climate. The improved HFACS-CM model is shown in Figure 2.

In the improved HFACS-CM model, the causes of accidents in deep coal mines are divided into five levels, including environmental factors, organizational influence, unsafe supervision, unsafe state of miners, and the unsafe acts of miners. Compared with the traditional HFACS model, the new model takes environmental factors as the first level and changes the precondition for the unsafe acts to the unsafe state of miners according to the real-world situation. According to the types of hazardous acts in the improved HFACS-CM model (26) and the actual conditions of coal mine production, this study defines the concept of unsafe acts of miners, as shown in Table 1.

**Table 1:** Types and contents of unsafe acts of miners.

**Types**	**Contents**
Decision errors	It is usually manifested as improper implementation of work procedures, risky operation and improper choice of risk response measures.
Skill-based errors	Usually manifested as a lack of safety knowledge, professional knowledge and basic operation skills, resulting in errors in the work.
Perceptual errors	It is usually manifested by the lack of safety awareness and poor self-protection and mutual protection awareness, for example, the failure to take safety protection measures when disasters occur.
Routine violations	It is usually manifested as the behavior in violation of rules and regulations, which is often a habit and not easy to be supervised and managed.
Exceptional violations	This usually manifests itself in the violation of specific regulations, such as those related to COVID-19.

### Grounded Theory

Grounded theory is a bottom-up analytical method based on experience and materials, which focuses on the treatment of problem situations and can produce solutions to problems (14, 29). This theory is a mature approach to exploring the nature of research, allowing concepts and categories to emerge naturally with greater objectivity. This theory has been successfully applied in some fields. For example, Lcaa et al. (30) analyzed the reasons for the shoddy work of some electricians by using the grounded theory. They suggested that electricians' work requires high cognitive ability. Chung et al. (31) also used grounded theory to analyze the health problems of middle school students using smart devices for learning. They concluded that if students' health problems were well monitored and managed, they could form the ideal smart-device use habits. In addition, Malakoutikhah et al. (14) analyzed the causes of Iranian workers' unsafe behaviors using grounded theory. They proposed that the factors influencing workers' unsafe behaviors could be divided into organizational, personal, and socioeconomic factors.

**Figure 3:**
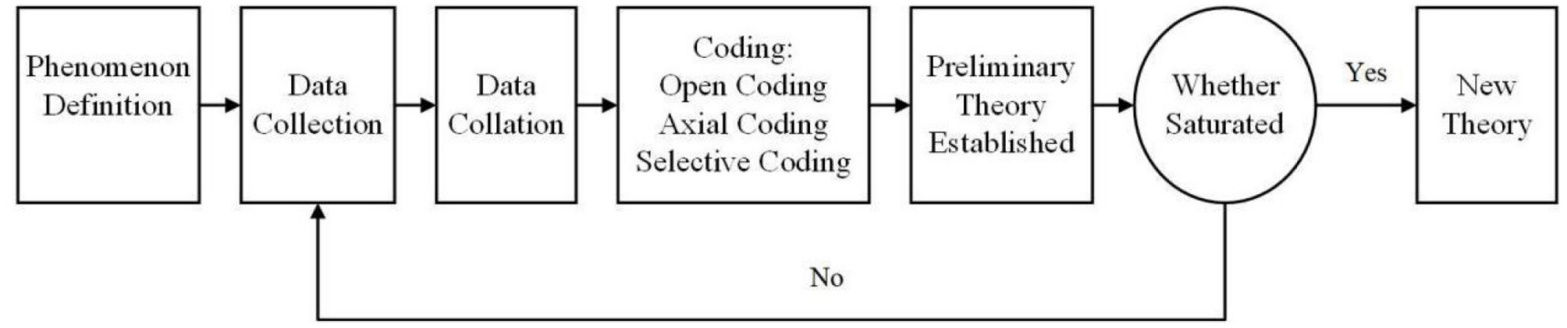
The process of grounded theory.

The analytical steps of grounded theory mainly include data acquisition, open, axial, and selective coding and model saturation testing, as shown in Figure 3. Open coding requires researchers to conduct an in-depth analysis of the original data with an open mind and code according to the state of the data. Open coding is divided into two stages: conceptual analysis and classification analysis. Conceptual analysis decomposes the interview data, and unsafe accident report data expresses the meaning of the original sentence in more refined sentences and conceptualizes the original data. The concepts obtained through concept analysis are scattered and similar, so they need to be connected to establish categories. Classification analysis refers to finding connections between the above concepts and further generalizing them into categories. Axial coding is the clustering analysis of the categories formed by open coding, and the correlation between different categories is established to create a larger category, namely the main category. Selective coding is based on axial coding, combing the relations between the main categories, abstracting the core categories that can summarize all categories, and illustrating the relations between the core categories and the sub-categories in the way of a story. A saturation test is needed to ensure the reliability and integrity of model construction. The original conceptual model is theoretically saturated if concepts or categories generated from newly collected data are incorporated into current concepts or categories, and no new concepts or categories are generated.

As shown in Figure 3, the research steps of the grounded theory include Phenomenon definition. Data collection. Data collation. Open coding, axial coding, and selective coding for data. Preliminary theory establishment. Test whether the theory is saturated. If the theory is saturated, a new theoretical framework will be formed. If the theory is not saturated, go back to step 2 and start again. The advantage of grounded theory is that it discovers, refines, and summarizes real-world problems from the bottom up based on experience and multiple sources. It is frequently used to study influencing factors (31, 32). Therefore, grounded theory based on data modeling is suitable for research to identify risk factors for unsafe acts in deep coal mine workers.

HFACS model and grounded theory are used together in this study to construct a conceptual model of risk factors for the unsafe acts of deep coal miners. First, based on grounded theory, we fully extracted coal miner workers' risk factors and types of hazardous actions of coal mine workers from coal mine accident reports and workers' interviews. Next, the factors were mapped according to the improved HFACS-CM model. Finally, the conceptual model of the risk factors of unsafe acts of deep coal mine workers was formed. In the process of extracting as many risk factors and types of hazardous acts of workers as possible, this study also analyzes the action path of these factors on unsafe acts and coal mine accidents.

## Results

### Data Collection

The range of data in grounded theory is so broad that everything is data (29). In other words, there is no limit to the types of data that could be examined, including textual materials such as literature, interview transcripts, accident reports, news, and other types of video and audio materials. Due to the limited literature on unsafe actions of deep coal miners, our study data comprises interview records and coal mine accident reports. These are textual data, which can be combined with and complement each other. Their combined use makes the conceptual model more comprehensive and consistent with the real-world conditions of a deep coal mine.

#### Interview Data Collection

Based on previous studies, this study designed an interview outline for risk factors of unsafe acts of deep coal mine workers from three aspects: influencing factors, unsafe behaviors, and their results, as shown in Table 2. We conducted one field survey and three online surveys from December 2021 to January 2022. The field survey unit was a deep coal mine in Huainan Area. The survey consisted of face-to-face interviews and one-to-one online video interviews, and the average interview time was 1 h. An on-site face-to-face interview was a primary way to collect interview data. The research object of this paper was the identification of the risk factors for hazardous action of deep coal mine workers, and these often occur in the production line. Therefore, the interviewees in this study are mainly ordinary employees, team leaders, and safety officers who are closely related to front-line production in coal mining enterprises. These employees understand unsafe acts and can provide a large amount of information. At the same time, to make the information obtained representative enough, the interviewees of this study are all from the lead majors in charge of front-line production. The interviewees included two middle-level leaders of the coal mine, three safety supervisors, three first-line team leaders, and 12 miners, altogether 20 people. In addition to asking questions according to the interview outline, the interviewees were also asked to talk about their views on the risk factors of unsafe acts according to their professional knowledge and work experience to obtain more research materials. Before the interview, we introduced the purpose and content of this survey. After getting the consent of the interviewee, we recorded the interview content. After the interview, the interviewees were numbered in Arabic numerals and edited into Word documents for further analysis.

**Table 2:** Interview outline.

**Outline numbers**	**Interview**
1	In your opinion, what factors may cause workers to have unsafe behaviors in the production process?
2	For the risk factors you list, can you point out how they contribute to the unsafe act of workers?
3	Can you point out the specific unsafe act of workers and managers at all levels in the daily production process?
4	What measures do you think can be taken to improve the unsafe act of workers?
5	What impact do you think the unsafe behavior of workers will have on the safety of coal payment?
6	In your opinion, from the perspective of coal payment safety, what measures should be taken to intervene in the unsafe act of workers?

The sociodemographic characteristics of respondents in this study are shown in Table 3. As shown Table 3, all interviewees are male, which also is determined by the particularity of mining work. The respondents were generally between 25 and 34 years old, accounting for 55% of the total respondents. Most respondents have a bachelor's degree or above, accounting for 60% of the entire survey population. Most respondents, 60 percent of the total, had worked for 3 years or less. Respondents with a monthly income of 5, 000 yuan or more accounted for more than 55 percent of the total respondents, and 60% of respondents were front-line employees, while the rest were managers at all levels. The vast majority of respondents were married, accounting for 70% of the total.

**Table 3:** Sociodemographic characteristics of interviewees.

**Survey Content**	**Item**	**Frequency**	**Percentage (%)**
Gender	Male	20	100
	Female	0	0
Age	18–24 years old	4	20
	25–34 years old	11	55
	35–44 years old	3	15
	45 years old and above	2	10
Degree	Junior high school and below	0	0
	A high school diploma	4	20
	College degree	4	20
	Bachelor's degree	10	50
	Master degree and above	2	10
Work time	1 year or less	2	10
	1–3 years (including 3 years)	10	50
	3–5 years	5	25
	5 years and above	3	15
Income	3,000 RMB and less	1	5
(RMB/month)	3,000–5,000 RMB (including 5,000 RMB)	8	40
	5,000–10,000 RMB	8	40
	10,000 RMB and above	3	15
Jobs	Middle management	2	10
	Safety supervisor	3	15
	First-line team leader	3	15
	Front-line workers	12	60
Marriage	Married	14	70
	Unmarried	6	30

#### Deep Coal Mine Accident Report Collection

**Figure 4:**
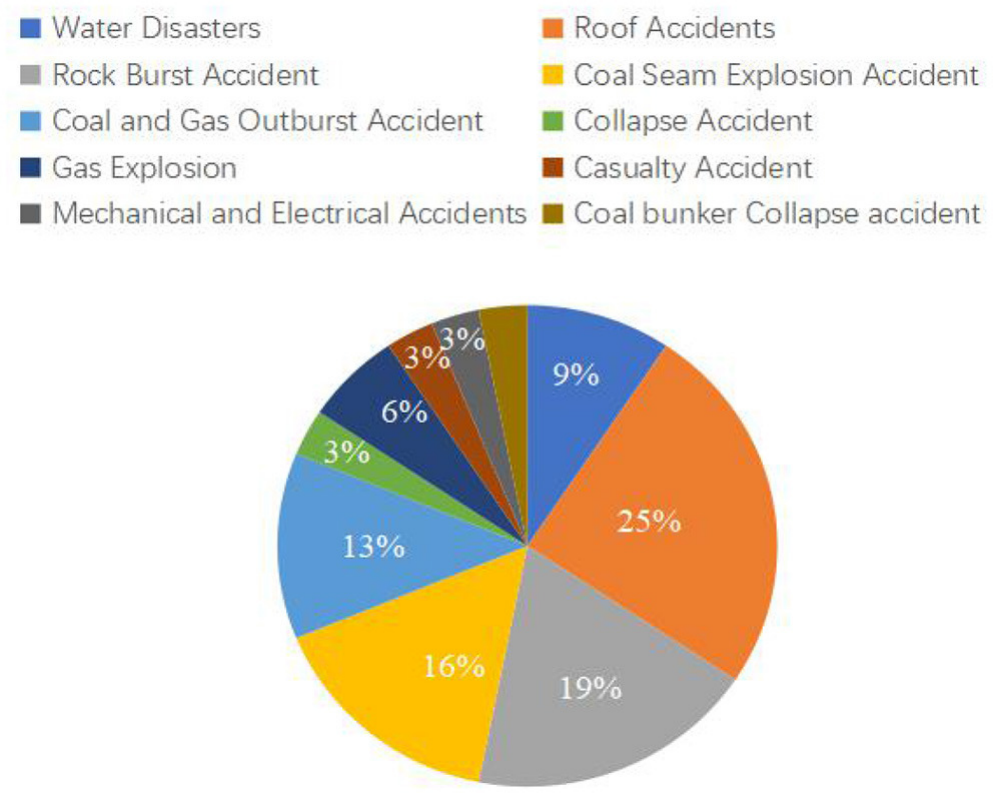
Statistics of coal mine accidents.

In grounded theory, original data is crucial for establishing new theoretical systems, and the information released by authoritative institutions is more reliable and accurate. Therefore, the coal mine accident report issued by China Coal Mine Safety Production Network is selected as the original data. This website has accumulated a large number of coal mine accident cases. These data contain detailed process descriptions and cause analysis of accidents, which apply to grounded theory analysis. Furthermore, the accident investigation report can reveal the cause of the accident in detail, the fault in the risk management process of the coal mine enterprise, and the defects of various constructive documents. Therefore, this paper uses accident investigation reports as the original data of grounded theory. Only the deep coal mine accident report with a mining depth over 600 m is selected in selecting coal mine accident reports. After screening, 40 accident reports from 2016 to 2021 are selected. The accident types mainly include ten major accident types, including rock-burst accidents, coal seam explosion accidents, water disasters, collapse accidents, mechanical and electrical accidents, coal and gas outburst accidents, roof accidents, and so on, as shown in Figure 4.

### Coding Process

#### Open Coding

Open coding requires researchers to conduct an in-depth analysis of the original data with an open mind and code the data according to the state of the data. Open coding is divided into two steps: conceptual analysis and classification analysis. The first step of conceptual analysis is to conceptualize the original data by disassembling the data and using more refined sentences to express the meaning of the original sentence. In this step, similar and identical statements in the original material are combined, some direct and vague statements are deleted, and only the statements related to the topic are retained. The second step of classification analysis is based on the core theme of risk factors of unsafe acts of deep coal mine workers, classifying and analyzing the types and risk factors of unsafe acts caused by interview data and accident reports sentence by sentence. This study combines the method of expert consultation with the technique of personal coding, reducing the number of personal subjective opinions. With the help of the expertise and experience of experts in the field, the subjectivity of coding is reduced. Nvivo 11 software is used to improve the scientific nature of the coding process. The coding materials were derived from 32 accident reports and 20 interview records. Through conceptual analysis and repeated arrangement and adjustment of original data, 327 concepts and 52 categories were abstracted. Then the similar categories of the 52 categories were combined to get 32 categories. This study obtained 52 secondary and 32 primary categories after open coding of the original data, as shown in Table 4.

**Table 4:** Summary of coding results.

**Item**	**Category**	**Open coding**	**Axial coding**	**Selective coding**
1	Natural causes	Natural facts	Physical environment	Environmental factors
2	Complex geological conditions			
3	Equipment lack	Mechanical defects	Technological environment	
4	Equipment failure			
5	Unreliable safety ventilation	Ventilation system defects		
6	Lack of automatic ventilation system			
7	Poor safety monitoring system	Incomplete safety monitoring system		
8	Lack of security monitoring system			
9	Weak government regulation	Lack of government oversight	Policy environment	
10	Lack of emergency drills	Lack of education and training	Resource Management	Organizational influences
11	Inadequate safety education			
12	Unreasonable personnel allocation	Unreasonable staffing		
13	Inadequate staffing levels			
14	Underinvestment of equipment	Underinvestment in safety		
15	Underinvestment			
16	Emphasize production over safety	Emphasize production over safety	Organizational climate	
17	Superpower mining	Superpower production		
18	Give out a superpower indicator			
19	Lack of communication between management levels	Lack of communication		
20	No communication during shift			
21	Lack of cooperation among team members	Lack of teamwork		
22	poor guidance of technical measures	Imperfect technical specifications	Organizational process	
23	Failure to modify technical measures in time			
24	Failure to modify the management system in time	Imperfect rules and regulations		
25	Lack of relevant regulations			
26	Failure to revise the operating procedures in time			
27	Lack of guidance on worker behavior	Lack of guidance	Inadequate supervision	Unsafe supervision
28	Failing to detect and stop illegal behaviors in time	Failure to intervene in unsafe behavior		
29	Poor supervision of violations			
30	Disorganization of working labor	Unreasonable labor organization	Planned operations	
31	Blind organization of production			
32	Disaster prevention and control measures are not	Poor risk management ability	Failed to correct problems	
	fully implemented			
33	Lack of Disaster response measures			
34	Poor risk assessment skills	Poor risk identification and assessment skills		
35	Poor identification ability of hazard sources			
36	Violation of operation procedures and requirements	Failed to enforce rules	Supervisory violations	
37	Illegal mining	Illegal business		
38	Fake information			
39	Operating personnel without a license			
40	Poor safety awareness	Poor safety awareness	Mental sates	Unsafe states of miners
41	Poor awareness of self-insurance and mutual insurance			
42	Lack of concentration	Lack of concentration		
43	Fatigue	Pressure of work		
44	Weak sense of responsibility	Lack of responsibility		
45	Avoid monitoring	Fluke mind		
46	Overburdened body	Physical fatigue	Physiological states	
47	Lack of basic safety knowledge	Lack of safety knowledge	Business ability	
48	Lack of disaster expertise	Lack of expertise		
49	Lack of basic protective skills	Lack of operational skills		
50	Adventure homework	Poor decisions	Errors	Unsafe acts of miners
51	Violation of rules and regulations	Acts against regulations	Violations	
52	Violation of laws and regulations			

#### Axial Coding

After open coding, we identified the main concept categories from 32 unsafe acts categories. The correlations between the main concept categories were established. These are the key content of axial coding. The process of axial coding further analyzed causal relationships and logical connections between categories obtained in open coding, and concepts that cannot be grouped with other concepts are deleted. Through the repeated comparisons and systematic clustering of 32 categories, 15 main categories were obtained, as shown in the third column of Table 4.

#### Selective Coding

Selective coding sorts out the relationships among the main categories, abstracts the core categories that summarize all categories and clarifies the relations between the core categories and sub-categories using a storyline. The core category is the thread of a fishnet, connecting all other categories. It serves as an outline. Through selective coding, five core categories of environmental factors, organizational influence, unsafe supervision, unsafe state of miners, and unsafe acts of miners were obtained, as shown in the fifth column of Table 4.

The storyline categorizes the risk factors influencing the unsafe actions of deep coal miners into four core categories: organizational influence, unsafe supervision, unsafe state, and environmental factors, which significantly impact the unsafe actions of miners. After analysis, the following storyline was be obtained. On the one hand, with the recent depletion of shallow coal mines, the depth of deep coal mines is increasing. The complex and changeable mine environment makes the mining of coal more difficult. It also makes supervision and administration of management departments difficult. Inadequate management of miners occurs due to the lack of oversight provided by management departments, weak risk awareness, imperfect supervision systems, and other reasons. For example, due to the unreasonable staffing of the management department, appropriate department leaders are frequently transferred to other positions. As a result, leaders are not focused and unwilling to manage the front-line miners, which may increase the likelihood of errors, violations, and other unsafe outcomes. On the other hand, the complex deep mine environment requires coal mining enterprises to increase investment in industrial processes, such as introducing advanced equipment, advanced technology, and hiring high-end talent. However, due to the lack of safety investment, the miners' skills and psychological quality will be significantly tested, greatly impacting worker performance and safety-related behaviors. Environmental factors affect organizational factors, organizational factors affect unsafe supervision, and unsafe supervision affects the worker conditions, thus affecting the workers' unsafe actions. The five core categories above dominate the other categories. They are ultimately summed up as a storyline that describes the risk formation process of unsafe behaviors of deep coal miners.

### Conceptual Modeling of Risk Factors

**Figure 5:**
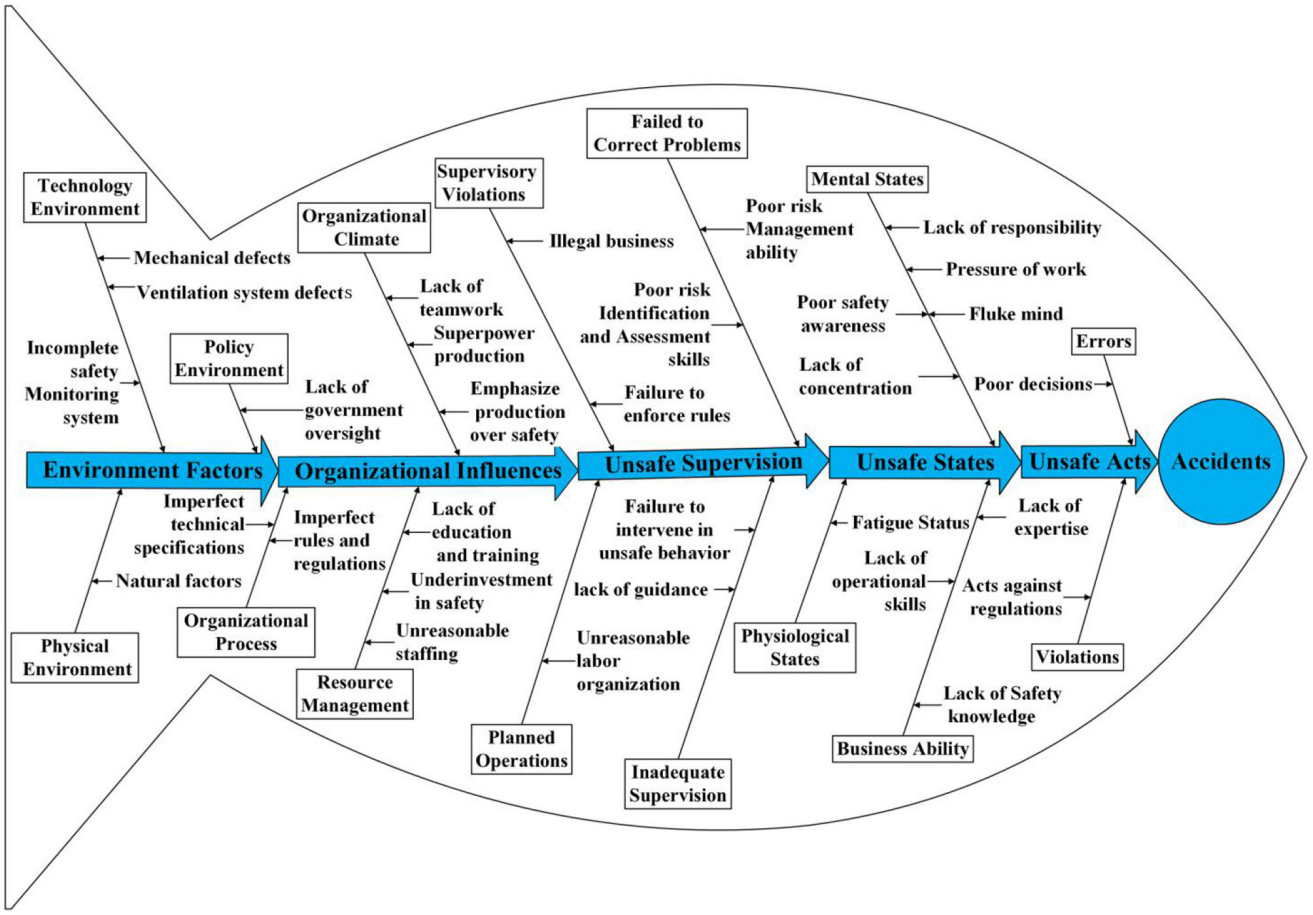
The conceptual model of risk factors of the miners' unsafe acts.

A conceptual model of risk factors for unsafe acts of deep coal mine workers is constructed by summarizing the coding results of accident reports and interview records, including five core categories and 32 sub-categories of factors, as shown in Figure 5. According to the story, environmental factors, organizational influence, unsafe supervision, and the unsafe state of the miners interact with each other, causing hazardous worker behaviors, mainly manifested as errors and violations, frequently leading to coal mine accidents.

The improved HFACS-CM model has similarities with many published HFACS models, but there are also some differences. First of all, the classification and description of the original HFACS model are relatively general. In contrast, the improved HFACS-CM model describes the risk factors of workers' unsafe acts at different levels according to the characteristics of the mining industry. Secondly, the hierarchy of human factors in the original HFACS model is divided. Still, the primary and secondary factors and the causal relationship between them cannot be identified. The improved HFACS-CM model elucidated the primary and secondary factors and the causal connection. Finally, the enhanced HFACS model analyzes human errors and violations at all system levels. This study has obtained a conceptual model of the risk factors of the unsafe act of workers in the deep coal mine, which has a more apparent conceptual relationship and hierarchical relationship.

### Model Saturation Test

A saturation test is needed to ensure the reliability and integrity of the conceptual model. If no new categories and logical relations appear in the original conceptual model by adding new data, the initial conceptual model is considered theoretically saturated. Otherwise, data collection must continue. The study's saturation test was performed 2 weeks after we formed the theoretical model. The saturation test was carried out by another member of the research group to eliminate the subjective influence of the researchers. The test data were eight previously prepared deep-coal mine accident reports. The test results showed that there were no new concepts. Furthermore, logical relations appear after the tests of the three coding processes of the grounded theory, indicating that the theoretical model previously constructed was saturated and there was no need to add data for analysis.

### Statistical Analysis of Risk Factors

**Figure 6:**
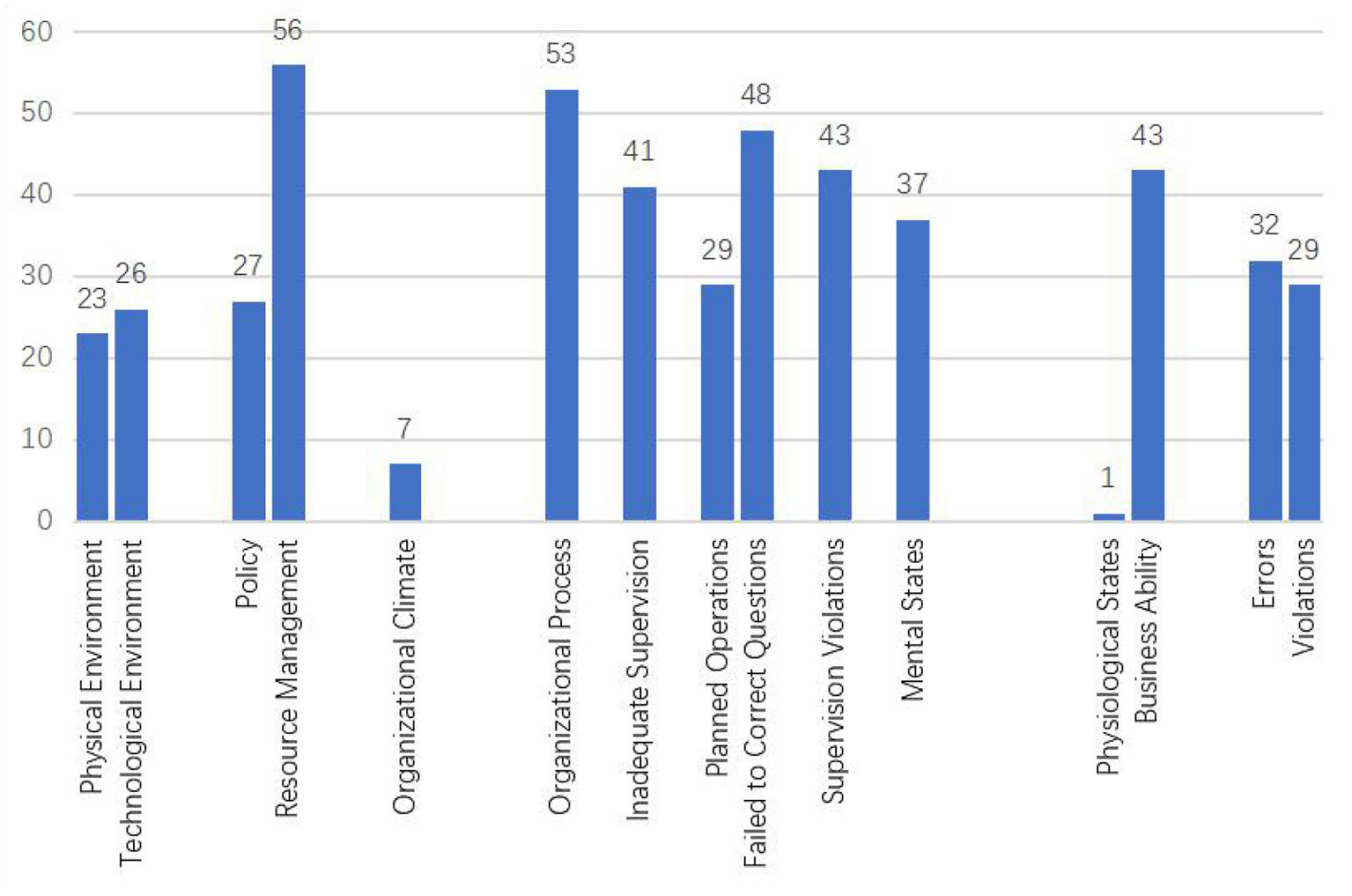
Statistics of influencing factors of unsafe acts.

Through statistical analysis of accident reports in deep coal mines, we obtained the frequency of risk factors of workers' unsafe acts, as shown in Figure 6. The top five risk factors for miners' unsafe acts are Resource Management (56), Organizational Processes (53), Failure to Correct Questions (48), Supervision Violations (43), and Business Ability (43), indicating that Resource Management is the most critical risk factor. Therefore, Resource Management should be strengthened to prevent and control hazardous actions of miners. The Influence of Unsafe Supervision (161), Organizational Influence (116), Unsafe State of Miners (81), and Environmental Factors (76) on the unsafe act of miners gradually decreases, which shows that Unsafe Supervision is the most important risk factor. In preventing and controlling coal mine workers' unsafe acts, managers should strengthen the supervision and management of workers' safety-related behaviors. Therefore, resource management and organizational process are the two most important risk factors affecting miners' unsafe acts, indirectly reflecting the importance of Organizational Influence. Organizational Influence is an indirect risk factor that affects workers' unsafe acts.

**Figure 7:**
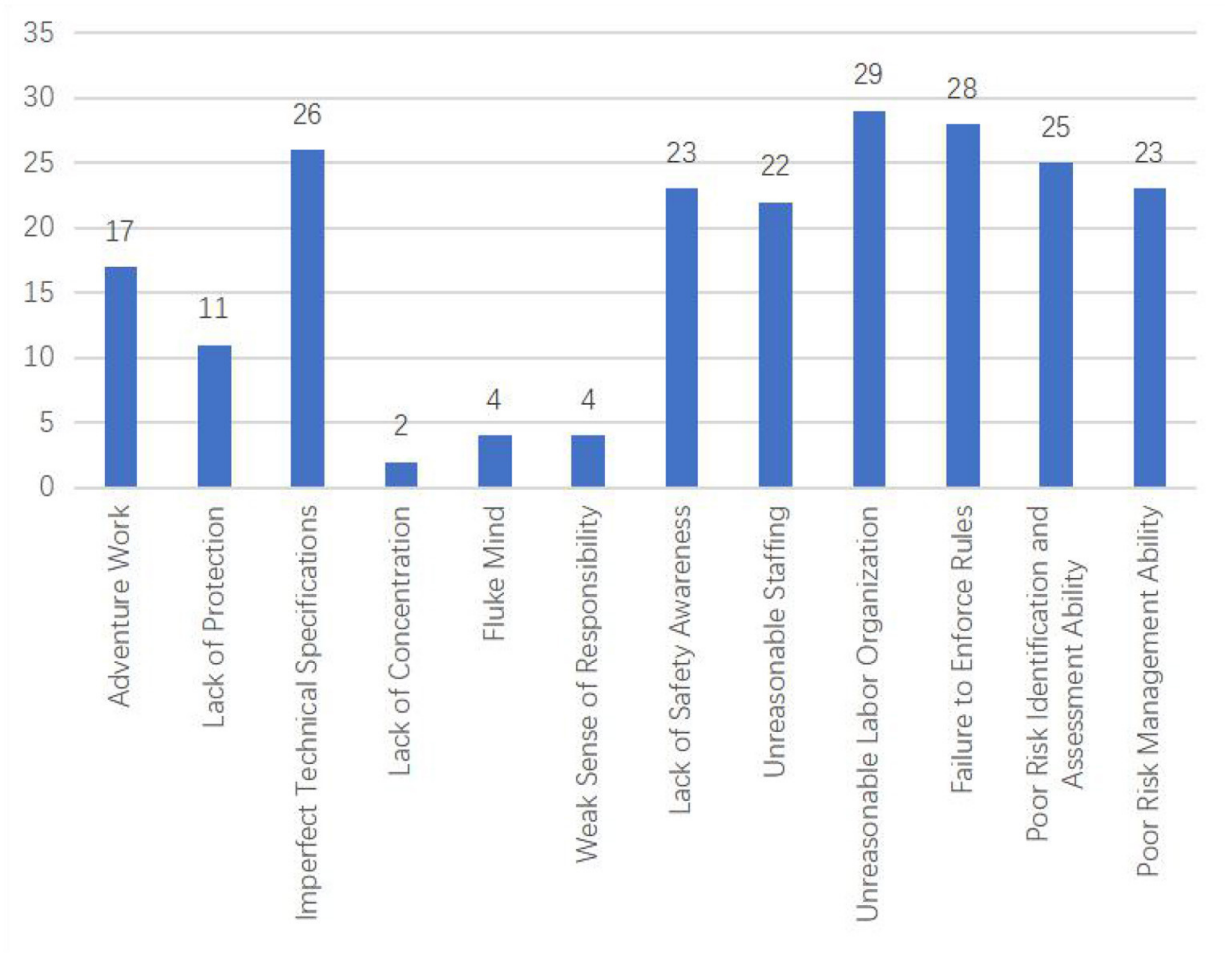
Statistics of miners' unsafe acts types.

According to the improved HFACS-CM model, the unsafe acts of deep coal mine workers are classified. These unsafe acts can be divided into five categories. They are Decision errors, Skill-based errors, Perceptual errors, Routine violations, and Exceptional violations. Decision errors include Adventure Work; Skill-based errors include Imperfect Technical Specifications and Lack of Protection; Perceptual errors include Lack of Safety Awareness, Lack of Concentration, Fluke Mind, and Weak Sense of Responsibility. Routine violations include Unreasonable Staffing, Unreasonable Labor Organization, and Failure to Enforce Rules; Exceptional violations include Poor Risk Identification and Assessment Ability and Poor Risk Management ability. Through statistical analysis of accident reports in deep coal mines, the frequency of various unsafe acts is shown in Figure 7. The frequency of violation act of deep coal mine workers is 127 times, and the frequency of error act is 87 times, indicating that the violation act of workers is higher. The most common violation was the Unreasonable Labor Organization, with 29, followed by Failure to Enforce Rules, with 28. The frequency of Imperfect Technical Specifications and Lack of Safety Awareness is 26 and 23 times, respectively, ranking first and second in all unsafe acts.

## Discussion

Based on the HFACS model and grounded theory, we established the improved HFACS-CM model. It was mapped by analyzing deep coal mine workers' accident reports and interview records. Finally, the conceptual model of risk factors for the unsafe acts of deep coal mine workers was established. Based on grounded theory, we concluded that risk factors affecting the unsafe actions of deep coal miners are mainly environmental factors, organizational influence, unsafe supervision, and unsafe state of miners. Among these factors, workers' unsafe state is direct, all others are indirect. The technique used in this study identified risk factors and systematically analyzed them.

### Theoretical Implications

The contributions of this study to coal mining safety include the following:

This research object of the study is deep coal mine workers. Our findings guide effectively addressing their unsafe behaviors. The unsafe acts of deep coal miners is the direct factor leading to coal mine accidents. However, previous research focused more on the miners' errors (5, 33), while research on regulatory violations of miners has been relatively limited, especially related to the unsafe actions of deep coal miners. Risk factors affecting the unsafe worker behaviors in deep mines have significantly increased compared to shallow ones. Previous studies on factors affecting the unsafe acts of miners are insufficient for analysis of unsafe behaviors of deep miners. Therefore, we should study the unsafe acts of deep miners. This study identifies their unsafe acts' risk factors and provides a theoretical basis for intervention strategies to prevent hazardous actions. Thus, our findings will help reduce the coal mine accidents caused by the unsafe acts of coal miners.

In this study, the HFACS model and grounded theory were used comprehensively, enriching the identification methods of risk factors for unsafe acts of miners and revealing the formation mechanisms of hazardous behaviors. The factors influencing coal mine workers' unsafe acts are complex and varied. Previous studies focused on unilateral factors such as fatigue, physical condition, and organizational defects (3–5, 24). In contrast, few studies have taken a systematic approach to analyzing the unsafe actions of miners from multiple perspectives. This study constructs a clear and comprehensive risk factor conceptual model for analyzing unsafe acts of deep coal miners from a systematic perspective. In addition, it clarifies the mechanism of risk factors on unsafe acts. Therefore, our findings provide a starting point for more detailed research in the future (34, 35).

This study provides a more profound and broader analysis of deep coal mine accidents. Although previous studies also statistically analyzed causes of accidents in coal mines from a human perspective (9, 36), they did not describe how various factors interact with each other. Based on its conceptual model of risk factors, our study provides a statistical analysis of types of unsafe acts and risk factors for miners, which indicates the degree of risk associated with each factor from a quantitative perspective. It also reveals the interactions between each factor. Therefore, the results provide a theoretical basis for future scholarly research (37).

### Practical Significance

The results provide valuable information for coal mine safety management practices.

Coal mine safety managers should pay attention to the complex factors contributing to workers' unsafe acts. They should work to reduce the occurrence of coal mine workers' unsafe behaviors from a systems perspective. The administration department of coal mine safety should take measures to intervene in the organizational factors that influence workers' behavior and in supervisory factors that impact working conditions. At the same time, managers should also consider the influence of these factors on workers' unsafe actions through their unsafe state. The conceptual model of risk factors constructed in this study shows that environmental factors, organizational factors, supervision factors, and workers' unsafe state interact, ultimately resulting in workers' unsafe actions. The coal mine safety managers can create safer conditions for workers and reduce the possibility of risky behaviors by improving the underground working environment, strengthening the organizational management, and enhancing supervision inside the mine.

Resource management for deep coal miners should be the critical content of unsafe acts intervention. It includes safety education and training, personnel allocation, and safety investment. Safety education and training often appeared among the risk factors of unsafe acts. For example, when education and training are incomplete, workers lack safety awareness and knowledge, which will affect workers' ability to identify and respond to risks (6, 8). Therefore, it is necessary to establish a sound safety education and training system at the organizational level. This strategy will strengthen safety education and training for workers and improve workers' safety awareness, knowledge, and operating skills, reducing the occurrence of errors and violations. In addition, due to the complexity of deep mining conditions, coal mining enterprises should increase investment and introduce advanced equipment, technology, and expert professionals to reduce the occurrence of unsafe acts of workers.

Supervision and preventative management of unsafe actions of coal miners also need to be strengthened. Based on the conceptual model of risk factors, Managers of coal mines can take the following measures to improve the supervision of workers' unsafe acts. First, strengthen the education and training of managers to let them timely guide miners' work and correct their unsafe acts. Second, improve legal standards and operating procedures to help workers reduce errors and violations. More detailed and comprehensive operational practices can help workers reduce the number of unsafe acts. More comprehensive rules and regulations would make it easier for managers to regulate miners' acts and reduce workers' violations. Finally, strengthen safety education and training for front-line miners, especially safety and professional knowledge. Rich professional knowledge and safety knowledge can enhance miners' safety awareness and professional skills and reduce the occurrence of safety acts.

Unreasonable labor organization and failure to enforce rules are the core risk factors of unsafe acts of deep coal mine workers. In the process of intervening in unsafe acts of miners, the manager should emphasize controlling these two factors. Reasonable labor organization is the result of making good operation plans. Therefore, coal mining enterprises need to introduce professional management personnel. Experienced managers can develop scientific, reasonable work plans but efficiently find loopholes in the management process, effectively preventing unsafe worker behavior. Managers of coal mines can adopt intelligent devices to monitor compliance on the job for those who fail to enforce rules. For example, Managers can use wearable devices to monitor workers' physical and psychological indicators to judge their condition.

## Conclusion

In this study, the HFACS model and grounded theory were used to analyze interview records and case reports of deep coal mine accidents to identify risk factors and manifestations of workers' unsafe actions. This comprehensive risk identification method can accurately find the factors implicit in the text data, which can investigate and analyze the risky behaviors of deep coal miners. The risk factor conceptual model constructed accurately describes the causes of unsafe acts of miners and provides a theoretical basis for safety improvements. Scholars can also apply the research methods in this paper to other fields to identify risk factors for workers' unsafe actions, such as crews and pilots.

There may be some limitations to this study. First, the conceptual model of risk factors constructed in this study was obtained by qualitative methods, and the influence of each factor on the unsafe act was not quantitatively analyzed. Based on this conceptual model, subsequent studies can quantitatively analyze the impact of various factors on workers' unsafe acts. In addition, the analysis process of grounded theory used in this paper was carried out manually with the help of Nvivo software. However, the amount of data collected and summarized manually is limited. Conducting manual analysis on large amounts of data is difficult. In the future, it should be considered to introduce data mining technology into the grounded theory to achieve rapid acquisition of crucial information from massive data and combine it with research content for coding.

This study provides a valuable basis and enlightenment for coal mine enterprises to supervise and manage unsafe behaviors of employees in practice and has practical guiding significance. Combined with occupational health-related laws and regulations and ISO 45001:2018, this paper puts forward some suggestions on the supervision and management of coal mine workers' unsafe behaviors from two aspects of control measures and supervision and management.

In terms of measures to control workers' unsafe behaviors, the risk factor identification model of coal mine workers' unsafe behaviors constructed in this paper can help managers of coal mine enterprises better understand the factors leading to coal mine workers' unsafe behaviors. According to the risk identification model, managers of coal mine enterprises can take measures to improve the unsafe situation of workers from three aspects: environmental factors, organizational influence, and organizational supervision, to reduce the occurrence of unsafe behavior. Improve the working environment for workers. Managers constantly improve and optimize the operating environment to eliminate and reduce workers' adverse psychological and physiological reactions caused by the negative environment. A good environment can make the operator work happily to avoid unsafe behavior. Strengthen safety education and training and enrich workers with safety knowledge. Safety education and training can continuously improve workers' safety awareness and skill quality to improve the overall quality of workers and reduce the occurrence of unsafe behaviors. Strengthen safety culture and create an excellent safe atmosphere to infect staff. Good safety culture and atmosphere can help workers establish safety concepts, make workers take the initiative to abide by rules and regulations and laws and regulations from the ideological point of view, and reduce unsafe behavior. Enterprises strengthen communication within the organization. Good communication within the organization can help employees solve various problems encountered in the work process to maintain a stable working mood and avoid unsafe behavior. Strengthen supervision and strictly restrict workers' behavior. Adequate on-site safety supervision is an essential means to standardize workers' safety behavior and ensure the implementation of safety systems and measures. Strict supervision and management of workers' safety behavior is the most direct way of effectively eliminating all kinds of unsafe behavior. Enterprises strengthen reward and punishment mechanisms. A perfect safety reward and punishment mechanism can timely punish workers' unsafe behaviors and effectively reward workers' outstanding performance, promoting workers to take the initiative to reduce the occurrence of unsafe behaviors.

The managers of coal mining enterprises can take the following measures to supervise and control the unsafe acts of coal miners. Establish and improve the mechanism for discovering, reporting, and appealing to workers' unsafe behaviors. Improve the education and evaluation system for workers with unsafe behaviors, and carry out professional intervention for workers with unsafe behaviors. Establish a system of returning and visiting workers who have performed unsafe behaviors to avoid the recurrence of unsafe behaviors. Conduct statistical analysis on various unsafe behaviors of workers to analyze the causes of unsafe behaviors and formulate effective control measures. Perfect the accountability system of workers' unsafe behavior to reasonably punish workers who have shown unsafe behavior.

## Data Availability Statement

The data analyzed in this study is subject to the following licenses/restrictions: Raw data can only be used by journal editors and reviewers, not publicly by others. Requests to access these datasets should be directed to Xue Wang, 102455019@qq.com.

## Author Contributions

XW designed and conceptualized the study, and wrote the manuscript. LY supervised the project and obtained funding. XW, JZ, and ZQ downloaded the related papers and also obtained funding. All authors participated in screening the articles and provided critical feedback, significantly contributed to the study, and approved the final manuscript.

## Funding

This study was supported by the National Natural Science Foundation of China under the grant (No. 71971003), the Major of National Social Science Foundation of China (No. 20ZDA084), and the Graduate Innovation Fund Project (No. 2020CX1009).

## Conflict of Interest

The authors declare that the research was conducted in the absence of any commercial or financial relationships that could be construed as a potential conflict of interest.

## Publisher's Note

All claims expressed in this article are solely those of the authors and do not necessarily represent those of their affiliated organizations, or those of the publisher, the editors and the reviewers. Any product that may be evaluated in this article, or claim that may be made by its manufacturer, is not guaranteed or endorsed by the publisher.
